# Design and Synthesis of Layered Na_2_Ti_3_O_7_ and Tunnel Na_2_Ti_6_O_13_ Hybrid Structures with Enhanced Electrochemical Behavior for Sodium‐Ion Batteries

**DOI:** 10.1002/advs.201800519

**Published:** 2018-07-01

**Authors:** Chunjin Wu, Weibo Hua, Zheng Zhang, Benhe Zhong, Zuguang Yang, Guilin Feng, Wei Xiang, Zhenguo Wu, Xiaodong Guo

**Affiliations:** ^1^ School of Chemical Engineering Sichuan University Chengdu 610065 P. R. China

**Keywords:** hybrid structures, reaction dynamics, sodium ion batteries, sodium titanates, synergetic effects

## Abstract

A novel complementary approach for promising anode materials is proposed. Sodium titanates with layered Na_2_Ti_3_O_7_ and tunnel Na_2_Ti_6_O_13_ hybrid structure are presented, fabricated, and characterized. The hybrid sample exhibits excellent cycling stability and superior rate performance by the inhibition of layered phase transformation and synergetic effect. The structural evolution, reaction mechanism, and reaction dynamics of hybrid electrodes during the sodium insertion/desertion process are carefully investigated. In situ synchrotron X‐ray powder diffraction (SXRD) characterization is performed and the result indicates that Na^+^ inserts into tunnel structure with occurring solid solution reaction and intercalates into Na_2_Ti_3_O_7_ structure with appearing a phase transition in a low voltage. The reaction dynamics reveals that sodium ion diffusion of tunnel Na_2_Ti_6_O_13_ is faster than that of layered Na_2_Ti_3_O_7_. The synergetic complementary properties are significantly conductive to enhance electrochemical behavior of hybrid structure. This study provides a promising candidate anode for advanced sodium ion batteries (SIBs).

Due to increasingly serious environmental pollution problems, renewable energy storage techniques had been proposed and rapidly developed to store green and clear energy sources, such as solar energy, wind energy, geothermal energy, tidal energy, and so on. Today lithium‐ion batteries (LIBs) have achieved a great development and success in these fields of electronic equipment, hybrid electrical vehicles (HEVs), and electrical vehicles (EVs).^1^, ^2^ However, with the increasing of market demand, the limited lithium resources and relatively high cost could severely restrict their large‐scale application.^3^, ^4^ As sodium could be abundant, harmless, and similar chemical properties to lithium, sodium ion batteries (SIBs) have been considered as a promising alternative to current LIBs. In the past few years, the anode materials as an important part of SIBs had attracted more attentions. And many anode electrode materials have been extensively studied, such as carbon‐based materials,^5^, ^6^, ^7^, ^8^ metal oxide compounds,^9^, ^10^, ^11^ alloy materials,^4^, ^12^ and titanium‐based materials.^13^, ^14^, ^15^ But there are still great challenges to prepare the efficient anodes of SIBs with higher energy efficiency, better cycling stability, and faster Na^+^ diffusion. Presently, sodium titanates materials reported as the anode electrodes of SIBs have attracted immense interest due to their efficient sodium ions storage activity, high cycling stability, low voltage plateaus and cost, and nontoxicity.^16^, ^17^ As a delegate of sodium titanates, Na_2_Ti_3_O_7_ had been reported to reversibly take up 2 Na ions per formula unit at an average potential (0.3 V vs Na/Na^+^).^18^ The low Na‐storage voltage not only resulted in a high energy density but also reduced the appearance of sodium dendrite.^19^ The layered Na_2_Ti_3_O_7_ structure was made up of zigzag type layer formed by linking and stacking TiO_6_ octahedra ribbon and delivered a high theoretical capacity (177 mAh g^−1^).^20^, ^21^ Nevertheless, one significant deficiency was that the insufficient conductivity, slow ionic mobility, and structural distortion upon Na uptake would severely hinder the development and application of Na_2_Ti_3_O_7_ anode in SIBs.^15^, ^22^ As another a delegate of sodium titanates, Na_2_Ti_6_O_13_ exhibited high ionic conductivity, long cycling stability, and low Na insertion/extraction potential. The tunnel channels could be far bigger than Na^+^ radius, which was conductive to the ionic mobility and resulted in a superior rate capability.^23^ The volume effect was accommodated and degradation of structure was avoided by the large interlayer, which resulted in the excellent cycling stability. Some previous work with Na_2_Ti_6_O_13_ as the anode of SIBs had presented efficient electrochemical properties of Na_2_Ti_6_O_13_.^24^, ^25^ But one major issue was that Na_2_Ti_6_O_13_ suffered from the low Na‐storage capacity. Based on the above description, whether there was one strategy of simultaneously accommodating the respective defects of single Na_2_Ti_3_O_7_ and Na_2_Ti_6_O_13_ structure and presenting a good electrochemical behavior or not. Of many studied strategies, some scholars provided that hybrid structures could effectively break the limitations of single structure and improve performance of SIBs, such as the layered‐spinel hybrid structures,^26^, ^27^ dual‐layered structures,^28^, ^29^, ^30^ layered‐tunnel hybrid structures,^31^, ^32^ tunnel–tunnel hybrid structures.^33^ Thus, one strategy with building layered Na_2_Ti_3_O_7_ and tunnel Na_2_Ti_6_O_13_ hybrid structure could be proposed. The expected result is that the hybrid structure could mutually offset respective defects of single structure and enhance Na‐storage behavior.

In this work, the novel layered Na_2_Ti_3_O_7_ and tunnel Na_2_Ti_6_O_13_ hybrid material (NNTO) was fabricated with a facile hydrothermal method and its Na storage properties were also evaluated. To confirm the constitution of hybrid structure, synchrotron X‐ray diffraction and powder X‐ray diffraction were performed. Moreover, in situ synchrotron X‐ray powder diffraction (SXRD) characterization was also carried out to elaborate its structural evolution and sodium ions insertion/desertion reaction mechanism for the first time. The synergetic effect between two phases and reaction dynamics of NNTO were also investigated in‐depth. The result indicated that NNTO consisted of the layered Na_2_Ti_3_O_7_ and tunnel Na_2_Ti_6_O_13_ component. In situ X‐ray diffraction (XRD) analysis demonstrated that the solid solution reaction occurred in tunnel Na_2_Ti_6_O_13_ component and phase transition appeared in the layered Na_2_Ti_3_O_7_ structure. The synergetic effect between two phases could effectively offset the defects of single structure and significantly improve Na storage behavior. The obtained dates from reaction dynamics analysis presented that the ionic mobility of tunnel structure was faster to that of layered structure and the enhanced diffusion‐controlled capacity contribution leaded to capacity increase during the initial stage. Based on current cognition and obtained result, the electrodes of hybrid structure would be a promising candidate for SIBs and applicable to the large‐scale energy storage systems in the future.

**Figure** **1**a,b exhibited the crystal structure of layered Na_2_Ti_3_O_7_ and tunnel Na_2_Ti_6_O_13_ along the *b* axis, respectively. Layered Na_2_Ti_3_O_7_ was assigned to *P*2_1_/*m* space group of monoclinic system and its lattice parameters were *a* = 9.133 Å, *b* = 3.806 Å, *c* = 8.566 Å, *V* = 291.7056 Å^3^, and β = 101.57°. In its unit cell, three TiO_6_ octahedra linked by edge‐sharing formed short ribbons, which shared their corners to form a zigzag (Ti_3_O_7_)^2−^ layer.^19^ Alkali metal ions were located at the middle of zigzag layers with occupying two different sites: Na1 and Na2 (Table S1, Supporting Information). Sodium ions were 9‐ and 7‐coordinated to oxygen atoms, respectively.^20^ Tunnel Na_2_Ti_6_O_13_ was attributed to the monoclinic *C*2/*m* space group. Sodium ions were residing in the interlayer with occupying one site: Na1 (Table S2, Supporting Information). Its cell parameters were *a* = 15.131 Å, *b* = 3.745 Å, *c* = 9.159 Å, *V* = 512.1783 Å^3^, β = 99.3°.

**Figure 1:**
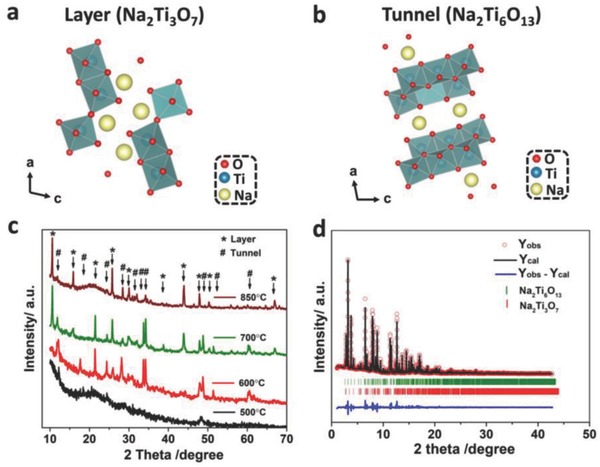
Schematic crystal structures: a) Layered Na_2_Ti_3_O_7_, b) tunnel Na_2_Ti_6_O_13_; c) XRD patterns of NNTO at the different temperatures; d) Rietveld refined SXRD (wavelength = 0.41237) curves of NNTO sintered at 850 °C.

As shown in Figure 1c, the product obtained by the hydrothermal reaction for 48 h at 180 °C was first annealed at 500 °C for 6 h with subsequent heat treatment at different temperatures for 12 h. The result showed that the optimal sintered temperature was 850 °C due to the better crystallinity. The constitution of NNTO samples was confirmed by XRD and SXRD analysis (Figure 1c,d) and all the diffraction peaks were well indexed to standard cards (JCPDS NO. 00‐031‐1329 and JCPDS NO. 01‐073‐1398). Rietveld refinement of SXRD patterns was carried out to identify the detailed structure characteristics by using Fullprof program. The result demonstrated that the percent of layered Na_2_Ti_3_O_7_ and tunnel Na_2_Ti_6_O_13_ was 26.22% and 73.78%, respectively (Table S3, Supporting Information). In addition, theoretical capacity of NNTO was calculated according to the theoretical capacity and percent of single component (Table S4, Supporting Information).

During the preparation process, different hydrothermal periods were explored with keeping other same condition (Figure S1, Supporting Information). Rietveld refinement of different XRD patterns was performed by using PDXL software. The relevant lattice parameters were portrayed in Table S5a–d (Supporting Information). The result revealed that layered content of NNTO was gradually going up with the increasing of hydrothermal durations, which was in good agreement with the variation of XRD peaks intensity in Figure S1 (Supporting Information). As shown in Figure S2 (Supporting Information), the cycling performance of samples of different hydrothermal durations was evaluated. This result indicated that the electrochemical behavior could be related with the layered percent. Figure S3a–e and Figure S4a–d in the Supporting Information showed the morphologies of anatase TiO_2_ and sodium titanates. The morphology evolution of sodium titanates went through a process, which developed from short nanorods, then to the mixture of nanorods and microrods, and finally evaluated to homogeneous microrods that showed high crystallization and uniform sizes.

**Figure** **2**a showed transmission electron microscopy (TEM) morphologies of the as‐prepared NNTO samples and two phases closely contacted together. The width of Na_2_Ti_6_O_13_ microrod was wider than that of Na_2_Ti_3_O_7_. By careful comparison, it was found that the widths of two phases were much wider than that of the previous published paper,^24^, ^34^, ^35^, ^36^ which may hinder the bulk Na^+^ diffusion and result in a pseudocapacitive phenomenon. The selected area electron diffraction (SAED) pattern of Na_2_Ti_6_O_13_ exhibited a monoclinic characteristic spot pattern and proved that the phase was single crystalline (Figure 2b). Its high‐resolution TEM (HRTEM) images provided a set of clear lattice fringes with the interplanar spacing of 7.46 Å corresponding to (200) plane (Figure 2c). Figure 5d presented the clear SAED image of layered structure along [030] direction. Figure 2e displayed that its HRTEM image and the lattice spacing was 2.17 Å, indexed to (311) plane. Klein et al.^37^ reported that synergetic effect existed and significantly improved the electrochemical behavior between close contact two phases. According to the different diffusion kinetics of two phases, more proportion of current was distributed to the material with faster kinetics and less proportion of current was utilized for the material with slower kinetics. Based on TEM images and cycling performance at the high current density, a synergetic effect may exist between two phases.

**Figure 2:**
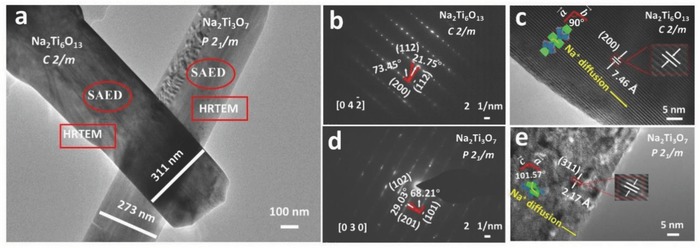
a) TEM images of NNTO; b,c) SAED and HRTEM of tunnel Na_2_Ti_6_O_13_; d,e) SAED and HRTEM of layered Na_2_Ti_3_O_7_.

The electrochemical performances of NNTO were evaluated by different characterizations. The cyclic voltammograms (CV) measurement at a scanning rate of 0.2 mV s^−1^ was conducted to investigate electrochemical behaviors of NNTO electrode. As depicted in **Figure** **3**a, a broad cathodic peak located at about 0.3 V was obviously observed and disappeared in the subsequent process. A pair of symmetrical redox peaks located at 0.74/0.93 V and one anodic peak located at 0.31 V could be clearly seen, which had been investigated to be corresponded to the reported characteristic redox pairs of tunnel Na_2_Ti_6_O_13_.^22^ For the discharge process, there was no other peaks to be observed when the discharge voltage exceeded 0.74 V. The behavior was in good agreement with solid solution phenomenon observed in situ XRD analysis. Another anodic peak located at 0.42 V could be also observed, and intensified and shifted toward a higher potential voltage (0.47 V), which was assigned to the redox pairs of layered Na_2_Ti_3_O_7_.^19^ The migration of the peak located at 0.42 V was related with sodium desertion of the layer structure, which was connected with phase transition of layer structural evolution. The CV curves could evidently overlap to result in better cycling performance after the 1st cycle. Figure 3b showed the discharge–charge curves of different cycling numbers at 20 mA g^−1^. The 1st discharge and charge capacity was 212.52 and 122.23 mAh g^−1^, respectively. The low Coulombic efficiency and huge irreversible capacity loss may be ascribed to the decomposition of electrolyte and the formation of solid state interphase (SEI) film. The discharge–charge profiles had multiple voltage plateaus and the electrode polarization was even severe with the cycling increasing, which was corresponding to the fall of capacity. Figure 3c showed the cycling performance of NNTO anode at a low current density. As compared to the electrochemical performance of single Na_2_Ti_3_O_7_ and Na_2_Ti_6_O_13_ phase (Figure S5, Supporting Information), the hybrid structures showed the excellent cycling performance and a high capacity. The cycling curve presented a characteristic that the reversible capacity increased gradually to a certain maximum value during the initial stage. To further explain why the capacity increased, CV measurements of different cycled NNTO samples at various scan rates ranging from 0.2 to 0.6 mV s^−1^ were performed (Figure S6, Supporting Information). The result indicated that the proportion of diffusion‐controlled Na‐storage was continuously improved and exhibited a linear increasing trend as compared to that of capacitive contribution with the cycling number increasing. And the capacity was rapidly decreasing, which may be caused by the larger volume effect from phase transition due to more Na^+^ insertion. Figure 3d exhibited that the NNTO electrode possessed a superior rate capability. Even the current density enhanced to 2000 mAh g^−1^, a reversible capacity of 19.45 mAh g^−1^ with 100% Coulombic efficiency can be retained after 4000 cycles (Figure 3e). The NNTO electrodes exhibited an excellent stability at a high current density due to the less amount of Na^+^ insertion in the layered Na_2_Ti_3_O_7_, which resulted in a small volume effect.

**Figure 3:**
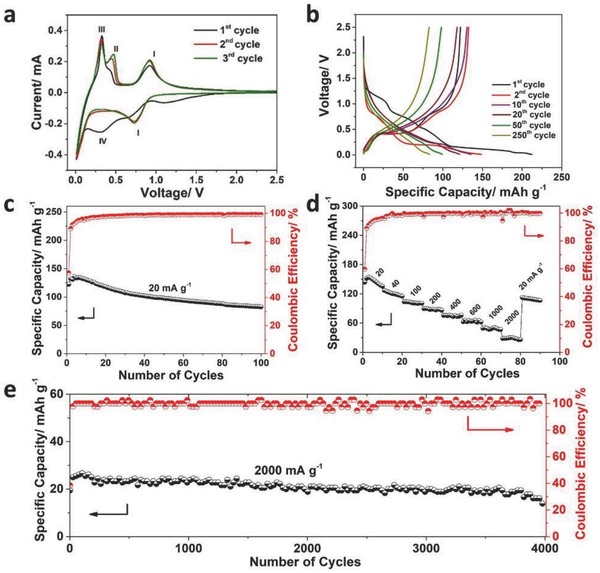
a) CV curves of a fresh electrode at a scan rate of 0.2 mV s^−1^; b) discharge–charge profiles of hybrid electrode at 20 mA g^−1^; c) cycling performance of the NNTO electrode at 20 mA g^−1^; d) rate capability at different current densities; e) the long cycling performance at 2000 mA g^−1^.

To investigate structural evolution of NNTO during the sodium ions insertion/extraction process, in/ex situ XRD measurements were carried out. As depicted in **Figure** **4**a and Figure S8 (Supporting Information), it was obviously observed that structural evolution was fully reversible due to reversible change of Bragg peaks. There was a solid solution reaction occurred in tunnel Na_2_Ti_6_O_13_, which was in accordance with the previous work.^38^, ^39^ It was worth noticing that SXRD characteristic peak located at 8.8° was splitting into two different peaks at the discharge state, which were incorporated again into one peak at the charge state (Figure S8, Supporting Information). This may be caused by the development of cell parameters.^40^ This phenomenon denoted that Na_2_Ti_6_O_13_ phase was positively taking part in the whole electrochemical reaction all the time. Based on in situ SXRD analysis, it was evident that phase transition occurred in layered Na_2_Ti_3_O_7_ when the 1st discharge voltage reached 0.11 V. Before phase transition, there was two phases coexistence for a short time around this potential, which implied that phase transition was a relatively rapid process. As shown in Figure 4a, the phases made up of Na_2_Ti_6_O_13_ and Na_2_Ti_3_O_7_ (NTO‐1) were converted into the mixed phases of Na_2_Ti_6_O_13_, Na_2_Ti_3_O_7_ and NaTi_1.25_O_3_ (NTO‐2), then developed into the compound of Na_2_Ti_6_O_13_ and Na_2_Ti_3_O_7_ (NTO‐3) at the discharge process vice versa. During the 1st charge stage, generated new phase was rapidly and reversibly turned into Na_2_Ti_3_O_7_ phase again. The Rietveld refinement of in situ SXRD patterns at a certain state was performed to further understand the hybrid structure characteristics (Figure S9a–d, Supporting Information). The lattice parameters produced from the refinement were listed in Table S6 (Supporting Information). With more Na^+^ inserting into the hybrid structure, new phase was evidently observed in Figure S9b (Supporting Information). By Rietveld refinement analysis, the new phase was detected to be tunnel NaTi_1.25_O_3_, which crystallized in the monoclinic system with *C*2/*m* space group and cell parameters were *a* = 21.555 Å, *b* = 3.7583 Å, c = 11.926 Å, *V* = 669.4282 Å^3^, and β = 136.14°. According to the result of Table S6 (Supporting Information), it was found that the cell volume expanded around 130% from phase transition, which might be responsible for the decay of capacity by a large margin by using pure Na_2_Ti_3_O_7_ anode as SIBs.^19^, ^22^, ^41^ In Figure S9c (Supporting Information), the new formed phase disappeared with Na^+^ extraction, which indicated that the structure was fully reversible between layered Na_2_Ti_3_O_7_ and tunnel NaTi_1.25_O_3_ phases . Based on ex situ XRD analysis (Figure S10a,b, Supporting Information), different NNTO electrodes at the different charge states showed that the peak intensity of active material decreased with the cycling increasing.

**Figure 4:**
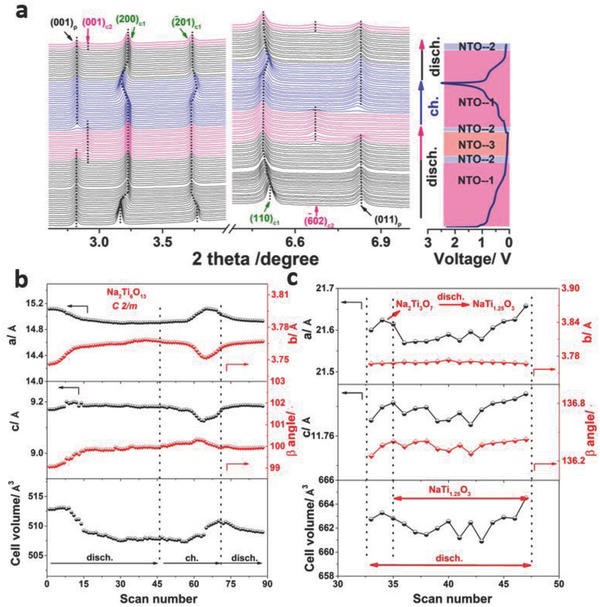
a) In situ SXRD patterns (wavelength: 0.41287 Å) in low angular regions and lattice planes of different phases contain different subscripts and colors: Na_2_T_3_O_7_ (subscript p and black color); Na_2_Ti_6_O_13_ (subscript c1 and olive color); NaTi_1.25_O_3_ (subscript c2 and pink color); the corresponding change of lattice parameters values for different components during the insertion/extraction process: b) Na_2_Ti_6_O_13_; c) NaTi_1.25_O_3_.

As showed in Figure 4b,c, Figure S11, and Table S7 in the Supporting Information, the lattice parameters values of respective component happened to alter, which was in accordance with the change of lattice plane. In terms of Na_2_Ti_6_O_13_ phase, 200_c1_ reflection was first shifted to high diffraction angles with Na^+^ insertion, then turned to lower diffraction angles with Na^+^ extraction, which was in agreement with the fluctuation of a parameter value. 110_c1_ and 020_c1_ reflections correlated with *b* parameter and −201_c1_ reflection corresponding to *c* parameter both showed the opposite trend. The change of Na_2_Ti_6_O_13_ cell volume varied in the range from 0.53 to 0.86%, which ensured the excellent cycling stability. It was especially noticeable that lattice parameters of Na_2_Ti_6_O_13_ phase had a slight change below 0.11 V of 1st discharge and 0.42 V of 1st charge. For Na_2_Ti_3_O_7_ component, there was significant lattice parameters change below 0.11 V of 1st discharge. For two‐phase transition region at the charge state, 020_p_ reflection shifted to higher diffraction angles, which referred to *a* decrease of *b* parameter value. The result of 011_p_ and 001_p_ reflections demonstrated a decrease of *c* parameter value. (−204_P_) reflection turned to lower diffraction angles, which implied an increase for a parameter value. For NaTi_1.25_O_3_ phase, its 001_c2_ and −602_c2_ reflections varied to the lower diffraction angles and the corresponding *c* and *a* parameter values increased.

As depicted in **Figure** **5**, schematic diagram of layered and tunnel structure evolution had been given by further analysis. Figure 5a showed the development of tunnel Na_2_Ti_6_O_13_ crystal structure without significant structural variation for Na^+^ insertion/extraction. With Na^+^ insertion developing, Na^+^ could first insert into 2d sites, then occupying 4i sites and further reaching 2c positions.^39^ Table S8a,b (Supporting Information) described the length variation of Na—O bonds of Na_2_Ti_6_O_13_ phase during the 1st discharge and charge process. In Table S8a (Supporting Information), the average Na–O distance was gradually decreasing from 2.69913 to 2.66408 Å after Na^+^ insertion. With the further inserting Na^+^, Na—O bond length slightly increased to 2.66474 Å. This may be resulting from the increasing repulsive Coulomb force between Na^+^. With Na^+^ extraction, the average Na–O distance was gradually going up. The bond valence sum (BVS) analysis has been carried out to evaluate the stability of Na^+^ insertion structure for tunnel Na_2_Ti_6_O_13_. The BVS values were calculated by two equations of *V*  =  Σ*v_i_* and *v_i_* =  exp[(*R*_0_ − *R_i_*)/*b*], where *R*_0_ = 1.803 and *b* = 0.37, *R_i_* is the length of Na—O bond.^39^, ^42^ As described in Table S9 (Supporting Information), the BVS values (around 0.323) were significantly smaller than the formal value of Na ion (1). This meant that Na^+^ could fit the interstitial position, which was advantageous to the stability of tunnel structure. Figure 5c presented the structural evolution of layered Na_2_Ti_3_O_7_ during the discharge–charge process. It was quite obvious that the structural development was reversible. Based on the BVS analysis and combined with the isosurfaces, Na^+^ migration path in tunnel and layered structure was visualized via VESTA software, respectively (Figure 5b,d). The result indicated that it was relatively easier for Na ions diffusion in the tunnel structure than that in the layered structure. Because the saw‐tooth pattern ions diffusion of layered structure produced the larger transmission resistance. Based on in situ SXRD results, schematic diagram of the synergistic mechanism was clearly observed. Due to the relatively faster diffusion dynamics, sodium ions could be first easier to insert into the tunnel structure. With sodium insertion proceeding, Na^+^ transfers to insert into the layered structure. For the charge stage, it is an opposite path due to the fully reversible structural evolution. The alternant insertion/desertion way means a remarkable synergetic effect. The faster diffusion dynamics of tunnel structure could offset the sluggish ionic migration of layered structure and the weak structural variation effectively relieves the huge volume effect from phase transition. The higher capacity of layered component could make up tunnel deficiency. The complementary properties of layered‐tunnel composite would be conducive to improve the respective defects and enhance the total electrochemical performance.

**Figure 5:**
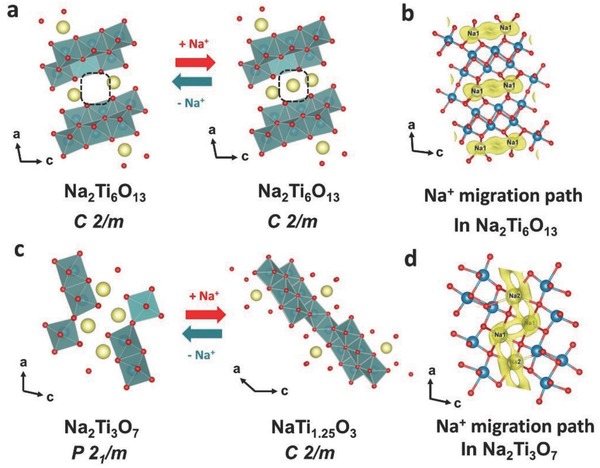
Schematic diagram of layered and tunnel structure evolution during the sodium ions insertion/extraction process: a) Na_2_Ti_6_O_13_ structure; c) Na_2_Ti_3_O_7_ structure; Na ions migration paths are simulated: b) tunnel Na_2_Ti_6_O_13_ phase; d) layered Na_2_Ti_3_O_7_ phase.

To investigate diffusion dynamics of different voltages, the galvanostatic intermittent titration technique (GITT) and voltage relaxation techniques were carried out. The battery was charging for 20 min and then relaxed 120 min for balance. The above process was continuously repeated until the 1st charge voltage reached 2.5 V. The diffusion coefficients were calculated according to the following equation^19^

(1) $$D_{\mathrm{GITT}}=\frac{4}{\pi \tau }\;{\left(\frac{m_{b}V_{m}}{M_{B}S}\right)}^{2}{\left(\frac{\Delta E_{s}}{\Delta E_{\tau }}\right)}^{2}$$

where, *D*_GITT_ is the Na^+^ diffusion coefficient, τ is the time period of current pulse during 1st charge stage at 0.2 mA g^−1^, *m*_b_ is the mass loading of the active material, *M*_B_ is the relative molecular weight, *V*_m_ is the molar volume, *S* is the area of electrode, Δ*E*_s_ is the difference of the open voltage at the end of two continuous relaxation period of steps, Δ*E*_τ_ is the difference of voltage at the beginning and end of the current pulse, *l* is the diffusion length of Na^+^.

As portrayed in **Figure** **6**a, the result indicated that the fluctuation of diffusion coefficient was corresponded to the change of the anode peaks of CV curve (Figure 3a). This denoted that tunnel Na_2_Ti_6_O_13_ had faster ion migration. To explore the reaction kinetics during the sodium ions insertion/desertion process, electrochemical impedance spectroscopy (EIS) was operated. The EIS result of the 1st, 10th, and 100th cycled NNTO samples was presented in Figure 6b and Figure S12 (Supporting Information). The solution resistance in the equivalent circuit (Figure 6b inset) is expressed by *R*_s_ *. R*_sei_ represents the SEI film resistance, *R*_ct_ denotes the charge transfer resistance, *Z*_w_ corresponded to the Warburg impedance. The equivalent circuit has been reported in the previous work.^34^, ^43^ As illustrated in Table S10 (Supporting Information), there is no evident variation for *R*_s_ at the different charge states. And the sum value of *R*_sei_ and *R*_ct_ is gradually increasing with the cycling increasing. But the impedance value is still small, which denotes a fast Na^+^ diffusion in the NNTO sample.

**Figure 6:**
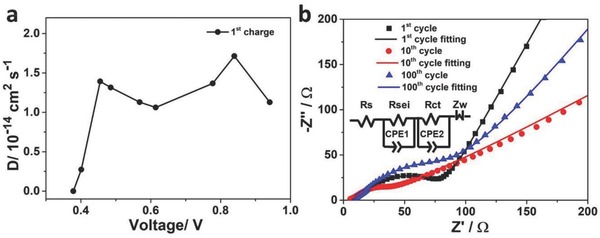
a) GITT curves at the 1st charge state at 0.02 mA; b) EIS plots of different cycled NNTO samples at different charge states (inset: equivalent circuit).

In summary, a new class of sodium titanates with layered Na_2_Ti_3_O_7_ and tunnel Na_2_Ti_6_O_13_ hybrid structures was fabricated by a facile hydrothermal method and applied as anode of SIBs. The hybrid material combined the advantage of layered Na_2_Ti_3_O_7_ with better Na‐storage capacity and tunnel Na_2_Ti_6_O_13_ with high ionic conductivity and excellent cycling stability. The complementary properties between both different structures resulted in excellent cycling stability and superior rate performance. The structural evolution and sodium ions insertion/desertion mechanism could be explored for the first time by using in situ SXRD analysis. The result exhibited that solid solution reaction occurred in tunnel Na_2_Ti_6_O_13_ component and phase transition appeared in layered Na_2_Ti_3_O_7_ structure. The structural evolution was fully reversible for single component during the discharge–charge process. The synergistic mechanism between two phases was also investigated in‐depth. The synergetic effect could effectively offset the defects of single structure and significantly improve Na‐storage behavior. The reaction dynamics of hybrid structures was further studied. The result indicated that tunnel possessed the superior rate capability. For this work, the novel sodium titanates could provide a new approach to design anode material for advanced SIBs.

## Conflict of Interest

The authors declare no conflict of interest.

## Supporting information

Supplementary
