# Supplementary material for: Design and Synthesis of Layered Na2Ti3O7 and Tunnel Na2Ti6O13 Hybrid Structures with Enhanced Electrochemical Behavior for Sodium‐Ion Batteries
Source: Adv Sci (Weinh). 2018 Jul 1;5(9):1800519. doi: 10.1002/advs.201800519 (PMC6145307; doi:10.1002/advs.201800519)
Supplement: Supplementary file 1 — Supplementary [file ADVS-5-1800519-s001.pdf]

Copyright WILEY-VCH Verlag GmbH & Co. KGaA, 69469 Weinheim, Germany, 2018.

ADVANCED SCIENCE
Open Access

Supporting Information

for *Adv. Sci.,* DOI: 10.1002/advs.201800519

Design and Synthesis of Layered $Na_2Ti_3O_7$ and Tunnel $Na_2Ti_6O_{13}$ Hybrid Structures with Enhanced Electrochemical Behavior for Sodium-Ion Batteries

Chunjin Wu, Weibo Hua, Zheng Zhang, Benhe Zhong, Zuguang Yang, Guilin Feng, Wei Xiang, Zhenguo Wu, and Xiaodong Guo**

Supporting Information

Design and Synthesis of Layered $Na_2Ti_3O_7$ and Tunnel $Na_2Ti_6O_{13}$ Hybrid Structures with Enhanced Electrochemical Behavior for Sodium-Ion Batteries

Chunjin Wu[1], Weibo Hua[2], Zheng Zhang[3], Benhe Zhong[1], Zuguang Yang[1], Guilin Feng[1], Wei Xiang[4], Zhenguo Wu[1,], Xiaodong Guo[1,]**

[1]School of Chemical Engineering, Sichuan University, Chengdu, 610065, PR China

[2]Institute for Applied Materials, Karlsruhe Institute of Technology (KIT), Hermann-von-Helmholtz-Platz 1, Eggenstein-Leopoldshafen 76344, Germany.

[3]College of Chemistry and Chemical Engineering, Xiamen University, Xiamen 361005, PR China

[4]College of Materials and Chemistry & Chemical Engineering, Chengdu University of Technology, Chengdu, 610059, PR China

Experimental Section

Materials: All the reagents used in the present study were obtained from Sinopharm and employed without further purification.

Synthesis of layered and tunnel hybrid material (NNTO): NNTO was prepared by a facile hydrothermal method. Anatase TiO_2 (0.5 g) was well dispersed into NaOH (2 M, 100 ml) solution by magnetic stirring about 40 minutes. Then the mixture solution was transferred into 150 ml Teflon-lined autoclave. The autoclave was maintained at 180°C for different hours in an oven and was taken out from the oven after cooling down to room temperature. The white precipitant could be obtained by centrifugation and washed to about pH 7 with deionized water. The obtained powder was dried at 80°C for overnight in a vacuum oven. Finally, this product was firstly sintered at 500°C for 6 h with subsequent heat treatment at different temperatures for 12 h. Heating rate of the total process was 5°C/min.

Morphology and phase analysis of NNTO: The morphology and crystallographic properties of the as-prepared samples were characterized by field emission scanning electron microscopy (SEM, HITACHI S-4800), transmission electron microscopy (TEM, JEOL 2100F), synchrotron radiation XRD (SXRD) at ALBA's beamline BL04-MSPD and powder X-ray diffraction (XRD, Panalytical EMPYREAN, Cu Kα radiation). The SXRD and XRD data was refined by Rietveld method using PDXL software (Rigaku Co., Ltd., PDXL 2.1) and FullProf program.

In situ X-ray characterization: In situ X-ray synchrotron diffraction (In situ XRD) measurement was operated at beamline P02.1 at the synchrotron diffraction instrument PETRA III (DESY, Hamburg). Detailed study of beamline P02.1 had been given by Herklotz et al. in 2013.[1] A 16-inch 2D flat panel detector of XRD 1621 N ES Series (PerkinElmer) with 2048 x 2048 pixels and a pixel size of 200 um was used for recording the diffraction

patterns.[2] In situ XRD data was collected in the 2θ range from 1.02° to 42.8°. The electrochemical test was performed during the 1st discharge down to 0.01 V and subsequent charge up to 2.5 V, then 2nd discharge down to 0.118 V and stopped. The XRD patterns were record one time every 10 mins interval. The cell battery was statically set for one hour when the 1st discharge process was finished.

Eletrochemical characterization of NNTO: The Electrodes were made by spreading a mixture of active material (hybrid materials), acetylene black and CMC binder with a weight ratio of 75: 15: 10 on copper foil current collectors, which were dried at 80°C for 12h in a vacuum oven. Electrochemical performances of the electrodes were evaluated by coin cells (type CR2025) assembled in an argon-filled glove box with O_2 and H_2O levels <0.5 ppm. For the half-cells preparation, sodium foil was used as the counter electrode and glass fiber (GF/D, Whatman) was used as the separator. The electrolyte was 1 M $NaClO_4$ and a mixture of ethylene carbonate/ propylene carbonate (EC: PC=1:1 v/v) with 2 wt % Fluoroethylene carbonate (FEC) (purchased from Fosai New Materials Co., Ltd., Jiangsu, China). The mass loading of active materials was around 2 mg cm^{-2}. The sodium cells were galvanostatically discharged and charged on a battery test system (Neware BTS-610) in a voltage range from 2.5 to 0.01 V (versus Na/Na^+). Cyclic Voltammetry tests were performed on an electrochemical workstation (LK 9805) in the voltage range of 2.5 to 0.01 V at a scan rate of 0.2 mV s^{-1}. The galvanostatic intermittent titration technique (GITT) tests were performed on a CT2001A LANHE electrochemical workstation. Electrochemical impedance spectroscopy (EIS) measurements were carried out by Zennium IM6 electrochemical workstation. Above all electrochemical measurements were conducted at 25°C.

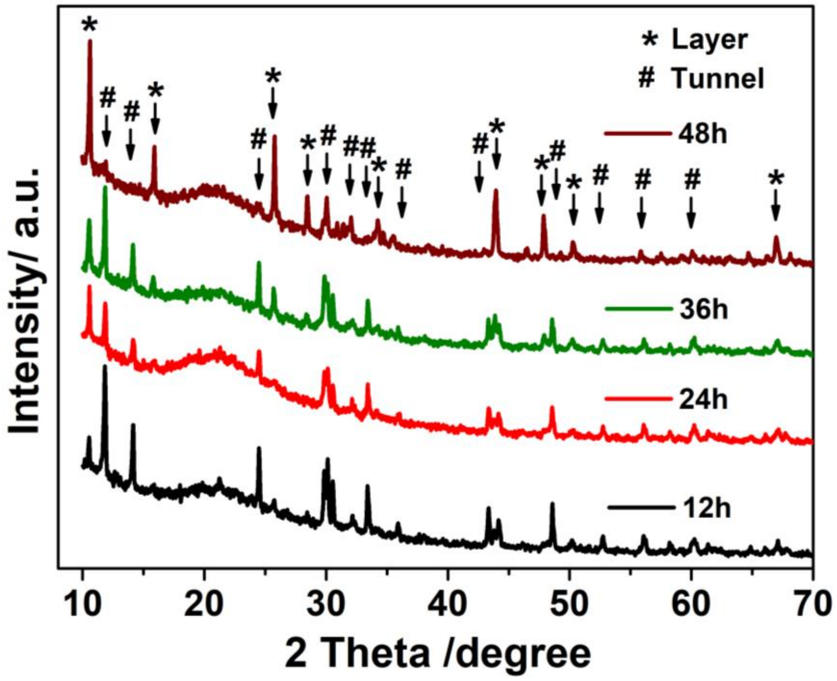

Figure S1. XRD patterns of samples in the different hydrothermal periods

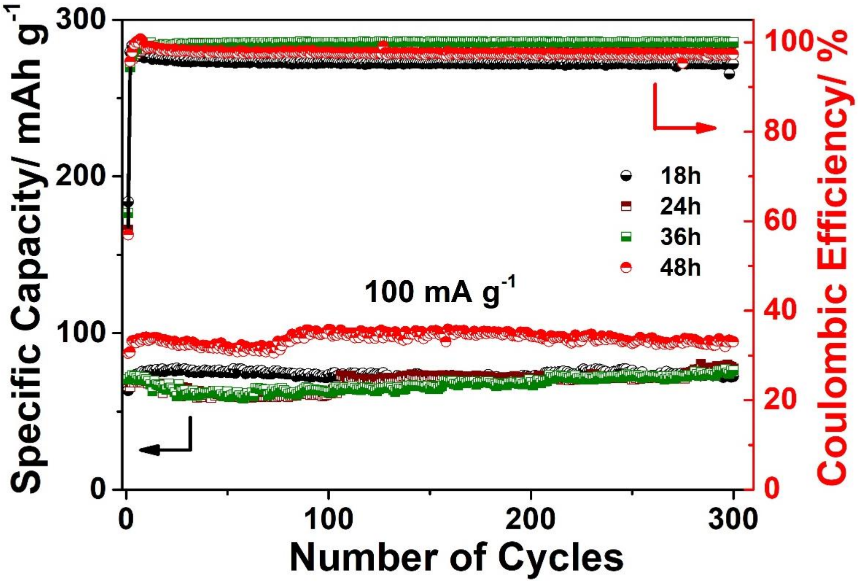

Figure S2. The cycling performance of samples for different hydrothermal durations at 100 mA g^{-1}

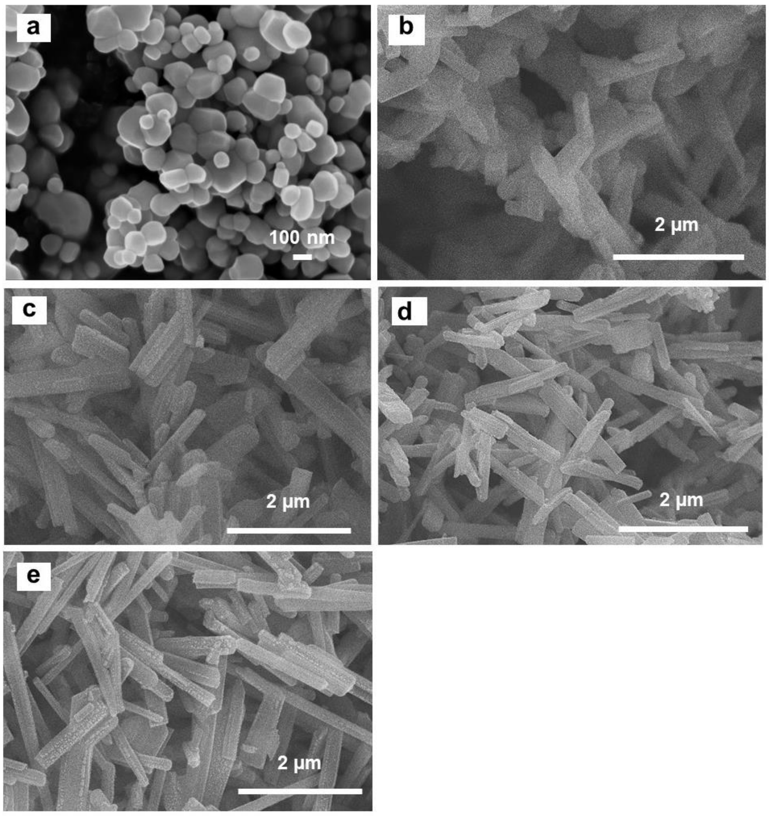

Figure S3. a) anatase TiO_2 particles; the high magnification SEM of samples for different hydrothermal time: b) 12h; c) 24h; d) 36h; e) 48h

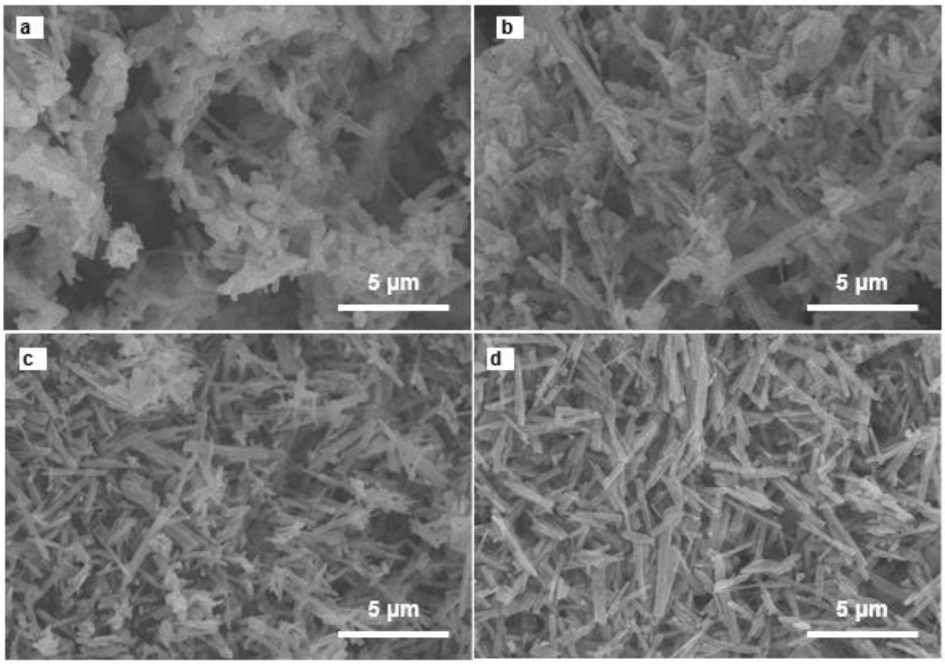

Figure S4. The the high magnification SEM of sodium titanates of different morphologies: a) 12h; b) 24h; c) 36h; d) 48h

To further clarify that the electrochemical performance of the hybrid structures could be superior to that of the pure $Na_2Ti_3O_7$ phase and $Na_2Ti_6O_{13}$ phase, the single $Na_2Ti_3O_7$ and $Na_2Ti_6O_{13}$ was fabricated by the transitional solid phase method. As shown in Figure S5, XRD analysis indicated that the single phase of sodium titanates was synthesized without impurity

phase. In addition, the cycling performance of single phase was detected at a current density of 20 mA g^{-1}. The result indicated that the large capacity decay was observed before 20 cycles for single $Na_2Ti_3O_7$ phase and the excellent cycling performance was given after 20 cycles. As showed in Figure S5 c, the capacity fluctuation of single $Na_2Ti_6O_{13}$ phase occurred with the poor cycling performance.

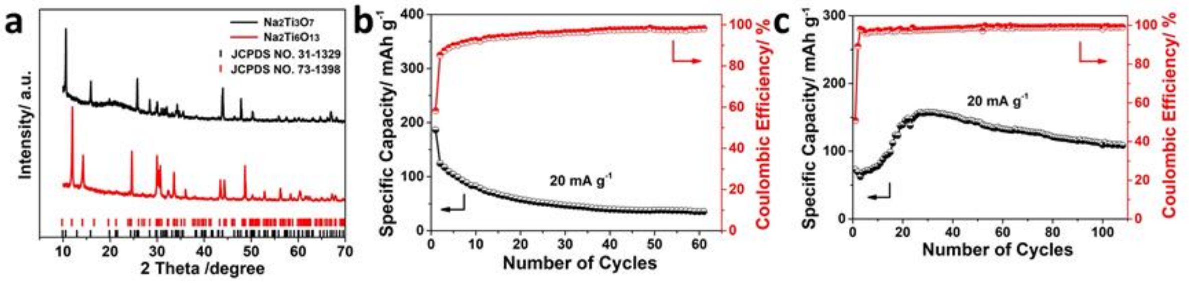

Figure S5. a) XRD patterns of single phase of $Na_2Ti_3O_7$ and $Na_2Ti_6O_{13}$; b) the cycling performance of $Na_2Ti_3O_7$ electrode at 20 mA g^{-1}; c) the cycling performance of $Na_2Ti_6O_{13}$ electrode at 20 mA g^{-1};

To further explain why capacity increase occurred during the initial stage, CV measurements of different cycled NNTO samples at various scan rates ranging from 0.2 to 0.6 mV s^{-1} were performed. The percent of capacitive/diffusion-controlled contribution of cycled NNTO at different charge states was presented in **Figure S6**. Based on CV curves of different charge states in Figure S6 (a, c, e), it was evidently observed that there were no obvious redox characteristic peaks of tunnel $Na_2Ti_6O_{13}$ of 10th cycled sample as compared to the 1st cycled sample. And the redox peaks of layered $Na_2Ti_3O_7$ located at around 0.11 V in Figure S5c was evidently seen, which was in good agreement with charge-discharge curves. With the cycling increase, the redox peak intensity of layered $Na_2Ti_3O_7$ at the 100th charge state became weaker and the redox peak intensity of tunnel $Na_2Ti_6O_{13}$ enhanced. The larger area space under CV profiles at the 10th charge state was much bigger as compared to the 1st and 100th cycled samples, indicating the better Na-storage. To confirm the surface/diffusion controlled contribution, the power law model is performed by the following equation：

$$\mathrm{i} = \mathrm{a}v^{b} \quad (\mathrm{S1})$$

Where a and b are adjustable values.[3, 4] The b value of 0.5 means that the current is

controlled by semi-infinite linear diffusion and the b value of 1 represents that the current is controlled by surface-controlled. As depicted in **Figure S7a**, the b value of the 1st cycle (0.791) exhibits that the Na-storage is mainly controlled from surface-controlled process. The b values of 0.568 and 0.572 at the 10th and 100th charge state suggests that the diffusion-controlled Na-storage occupies significant roles. Another analysis is also employed to confirm the percentage of surface/diffusion-controlled contribution and the equation is expressed as below:

$$\mathrm{I(V)} = k_1 v + k_2 v^{\frac{1}{2}} \tag{S2}$$

The above equation could be rearranged into another way:

$$\mathrm{I(V)}/v^{\frac{1}{2}} = k_1 v^{\frac{1}{2}} + k_2 \tag{S3}$$

Here $k_1 v$ and $k_2 v^{\frac{1}{2}}$ could represent capacitive and diffusion-controlled contribution.[5] Figure S7b shows the relationship of $i/v^{1/2}$ vs $v^{1/2}$ at different charge states. As portrayed in Figure S6 (b, d, f), the diffusion-controlled Na-storage is continuously improved and exhibits a linear increasing trend and reaches a maximum value at the 100th charge state as compared to that at the 1st and 10th cycled sample. This reason is that more active material takes part in redox reaction with more electrolyte penetrating from the outer surface to the bulk. The decay of capacity of 100th cycled sample may be related with the larger electrode polarization and the structural destruction of layered $Na_2Ti_3O_7$ from volume effect and the structural reconstruction/relaxation.[6, 7]

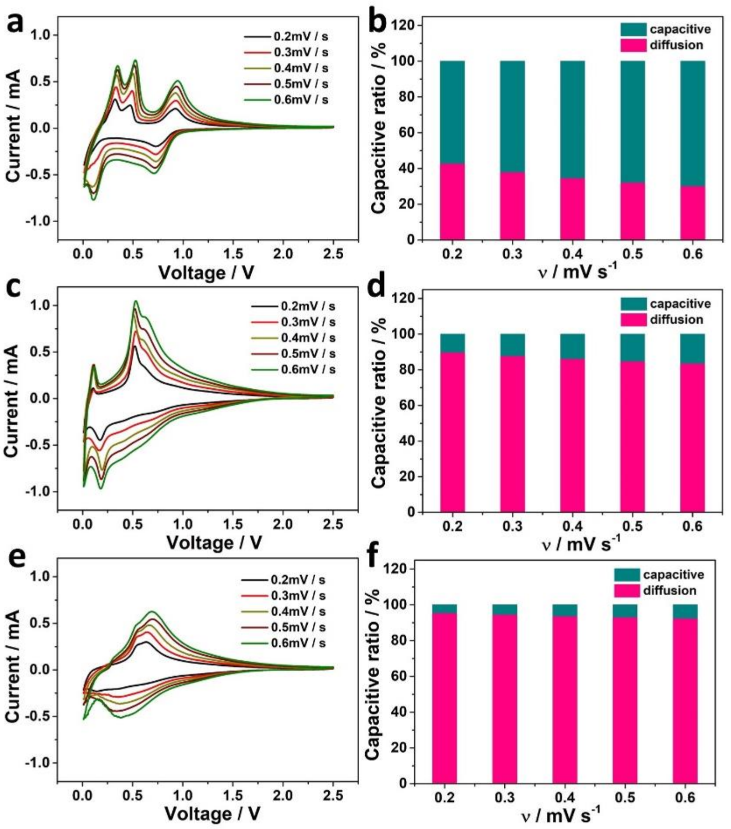

Figure S6. CV profiles of different cycled NNTO samples at different scan rates at the charge state: a) the 1st cycle, c) the 10th cycle, e) the 100th cycle; the ratio of capacitive/diffusion controlled contribution at the different charge states: b) the 1st cycle, d) the 10th cycle, f) the 100th cycle

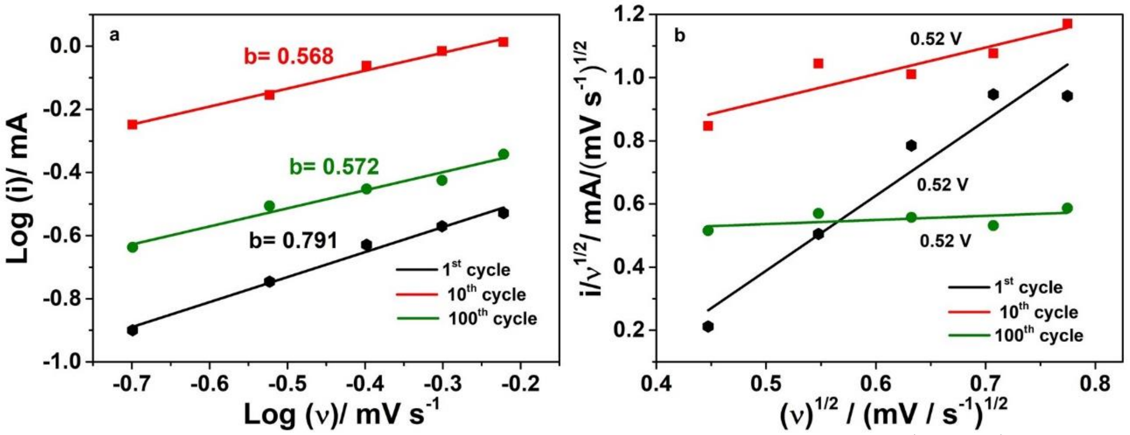

Figure S7. a) log (i) vs log (ν) curves and b) the curves of $i/\nu^{1/2}$ vs $\nu^{1/2}$

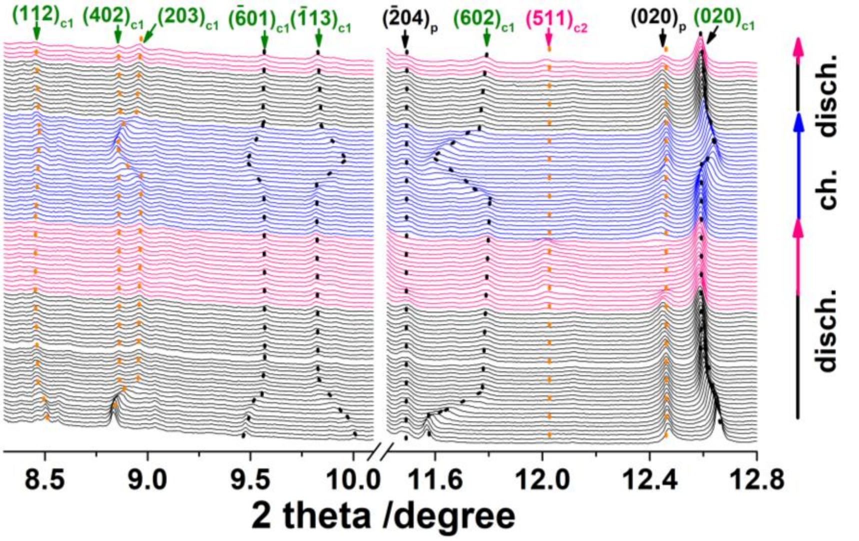

Figure S8. In situ XRD patterns of NNTO in high angular regions

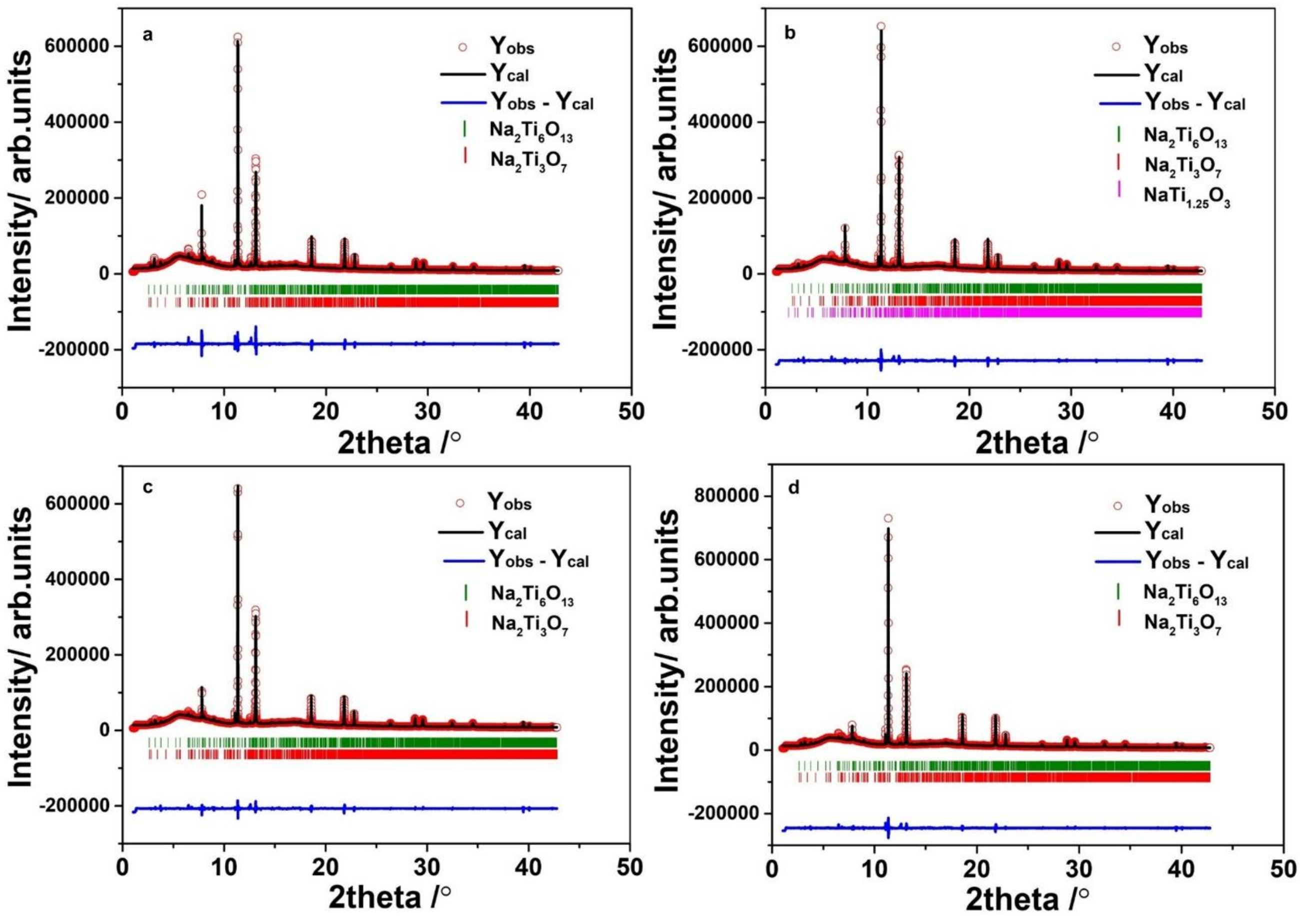

Figure S9. Rietveld refined of in situ XRD patterns at a certain state: a) 1st discharge to 1.063 V b) 1st discharge to 0.11 V; c) 1st charge up to 0.368 V (OCV voltage of 1st charge: 0.209 V); d) at the end of 1st charge

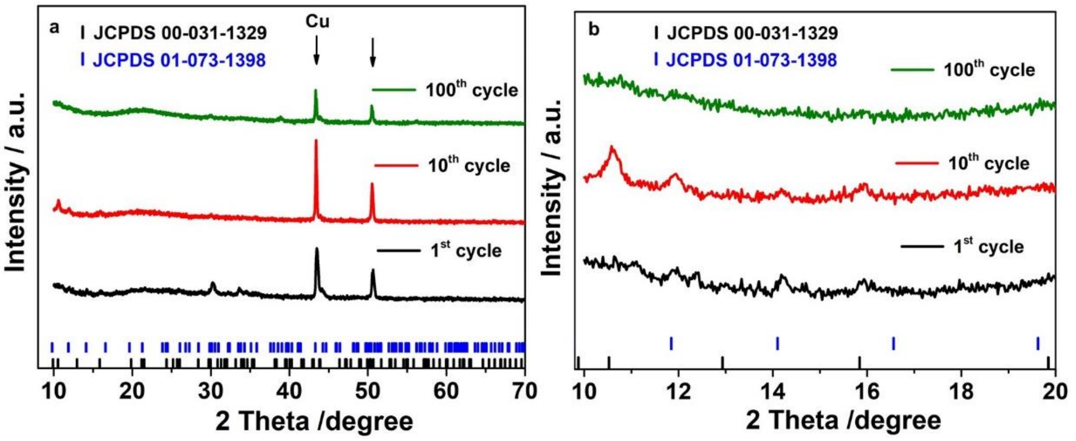

Figure S10. a) Ex situ XRD analysis of cycled NNTO samples at the different charge states; b) the extended Ex situ XRD patterns in low angular regions

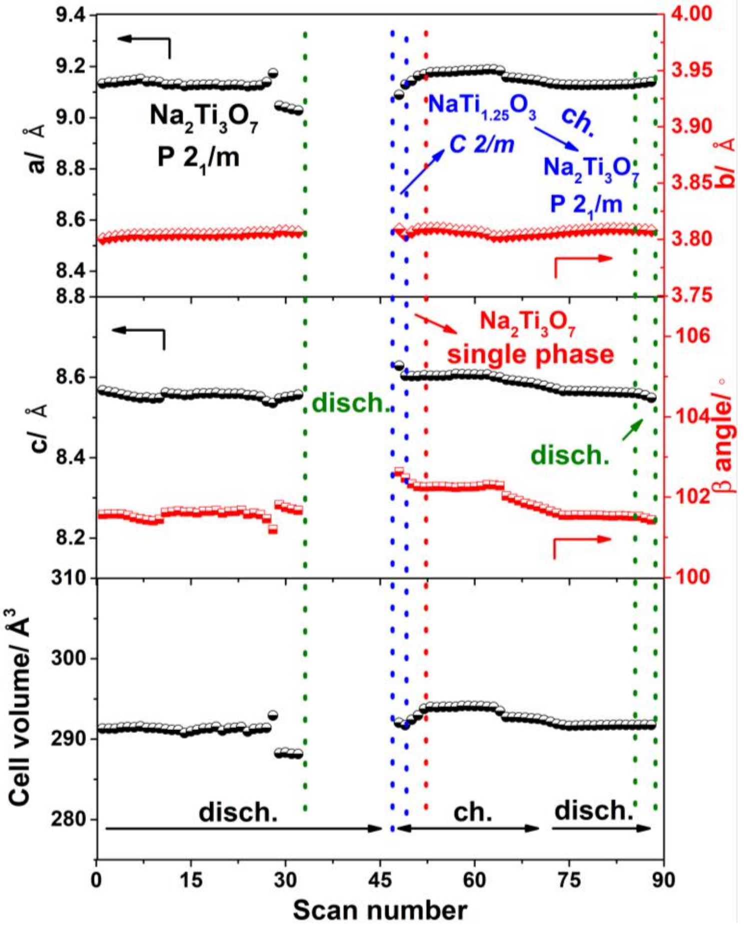

Figure S11. The corresponding change of lattice parameters values for different components during the insertion/extraction process

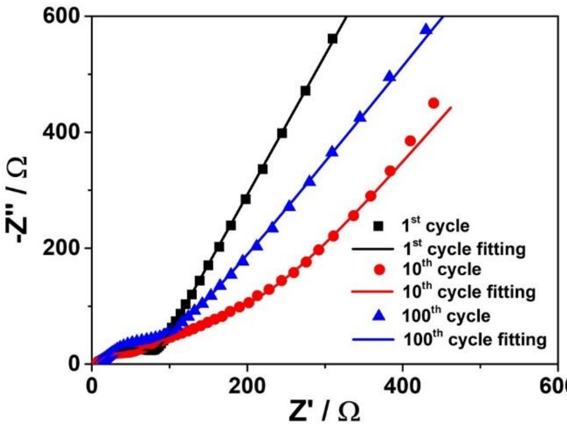

Figure S12. EIS plots of different cycled NNTO samples at different charge states

Table S1 The atom sites in layered $Na_2Ti_3O_7$

Atom	x	y	z	Occ	Wyckoff
Na1	0.5929	0.25	0.683	1	2e
Na2	0.1553	0.25	0.4999	1	2e
Ti1	0.98502	0.25	0.14606	1	2e
Ti2	0.67593	0.25	0.24887	1	2e
Ti3	0.2803	0.25	0.02857	1	2e
O1	0.1865	0.25	0.2188	1	2e
O2	0.4612	0.25	0.1465	1	2e
O3	0.651	0.25	0.4413	1	2e
O4	0.9081	0.25	0.3246	1	2e
O5	0.7532	0.25	0.0161	1	2e
O6	0.3182	0.25	0.8035	1	2e
O7	0.044	0.25	0.9152	1	2e

Table S2 The atom sites in tunnel $Na_2Ti_6O_{13}$

Atom	x	y	z	Occ	Wyckoff
Na1	0.454	0.00	0.2508	1	4i
Ti1	0.1137	0.00	0.0895	1	4i
Ti2	0.1705	0.00	0.4332	1	4i
Ti3	0.2287	0.00	0.7726	1	4i
O1	0.00	0.00	0.00	1	2a
O2	0.228	0.00	0.247	1	4i
O3	0.071	0.00	0.291	1	4i
O4	0.282	0.00	0.574	1	4i
O5	0.124	0.00	0.617	1	4i
O6	0.358	0.00	0.884	1	4i

O7	0.167	0.00	0.927	1	4i

Table S3 The result of Rietveld refinement of SXRD patterns

Space group	a [Å]	b [Å]	c [Å]	β [°]	Volume[$Å^3$]	R_{wp}	R_p	χ^2	Percent
C2/m	15.0974	3.7449	9.1723	99.02	512.19	7.33	5.15	8.31	73.78
P 2_1/m	9.1424	3.7955	8.5683	101.5793	291.272				26.22

Table S4 Theoretical capacity of NNTO by calculation

	$Na_2Ti_6O_{13}$ C2/m	$Na_2Ti_3O_7$ P 2_1/m	$Na_2Ti_6O_{13}$ C2/m	$Na_2Ti_3O_7$ P 2_1/m	NNTO
Theoretical capacity/ mAh g^{-1}	99	178	73.04	46.67	119.71
Percent/ %	100	100	73.78	26.22	100

Table S5a Rietveld refinement parameters of NNTO with hydrothermal reaction for 12h

	a [Å]	b [Å]	c [Å]	β [°]	Volume[$Å^3$]	R_{wp}	R_p	χ^2	Percent
C2/m	15.103	3.7452	9.1705	99.036	512.29	9.61	6.9	1.9119	95
P 2_1/m	9.386	3.8	8.701	102.08	303.5				5

Table S5b Rietveld refinement parameters of NNTO with hydrothermal reaction for 24h

	a [Å]	b [Å]	c [Å]	β [°]	Volume[$Å^3$]	R_{wp}	R_p	χ^2	Percent
C2/m	15.144	3.7482	9.1669	99.3	513.5082	5.19	3.82	0.8997	81
P 2_1/m	9.1066	3.795	8.541	101.57	289.1863				19

Table S5c Rietveld refinement parameters of NNTO with hydrothermal reaction for 36h

	a [Å]	b [Å]	c [Å]	β [°]	Volume[$Å^3$]	R_{wp}	R_p	χ^2	Percent
C2/m	15.095	3.7453	9.171	99.009	512.12	6.68	5.07	1.2824	74
P 2_1/m	9.146	3.8021	8.578	101.50	3				26

Table S5d Rietveld refinement parameters of NNTO with hydrothermal reaction for 48h

	a [Å]	b [Å]	c [Å]	β [°]	Volume[$Å^3$]	R_{wp}	R_p	χ^2	Percent
C2/m	15.31	3.694	8.67	100.6	482	5.4	4.06	0.9397	66
P 2_1/m	9.1309	3.8023	8.5706	101.58	291.5				34

Table S6 Rietveld refinement parameters of in situ XRD patterns at a certain state

	a [Å]	b [Å]	c [Å]	β [°]	Volume[$Å^3$]	R_{wp}	R_p	Rexp	State
C 2/m	15.117	3.746	9.1712	99.062	512.908	4.32	2.21	0.73	1.063V
P 2_1/m	9.133	3.8003	8.5678	101.566	291.339				disch.

C 2/m	14.9075	3.766	9.1870	100.00	507.944				0.11V
P 2_1/m	9.0824	3.8004	8.569	102.6979	288.552	4.13	2.44	0.77	disch.
C 2/m	21.599	3.7665	11.7811	136.2528	662.775				
C 2/m	14.9158	3.7663	9.1782	100.0146	507.758	4.10	2.42	0.75	0.368V
P 2_1/m	9.1300	3.8035	8.6037	102.4692	291.727				ch.
C 2/m	15.0324	3.7611	9.1660	99.9486	510.445	4.35	2.62	0.76	2.5 V
P 2_1/m	9.1400	3.8050	8.5791	101.7066	292.158				ch.

Table S7 The corresponding lattice parameters change of different components at different voltage regions

1st disch. Voltage/V		2.3-0.11	0.11-0.07	1st ch. Voltage/V	0.21-0.42	0.42-2.5
	a	↓	—		—	before↑after↓
$Na_2Ti_6O_{13}$	b	↑	↑		↓	before↓after↑
C 2/m	c	↑	—		↓	before↓after↑
	β	↑	—		↑	before↑after↓
	V	↓	—		—	↑
	a		↑			
$NaTi_{1.25}O_3$	b		—			
C 2/m	c		↑			
	β		—			
	V		↑			
	a	—			↑	↓
$Na_2Ti_3O_7$	b	—			↑	—
P 2_1/m	c	—			—	↓
	β	—			↓	↓
	V	—			↑	↓

Table S8a The length change of Na-O bonds of tunnel $Na_2Ti_6O_{13}$ phase during the 1st discharge process

State	2.3 V [Å] (1st discharge)	1.063 V [Å] (1st discharge)	0.11 V [Å] (1st discharge)	0.054 V [Å] (1st discharge)
Na1-O3	2.56213	2.47784	2.46774	2.46861
Na1-O5	2.61313	2.63281	2.63977	2.63965
Na1-O7	2.92314	2.90647	2.88473	2.88596
Average length	2.69913	2.67237	2.66408	2.66474

Table S8b The length change of Na-O bonds of tunnel $Na_2Ti_6O_{13}$ phase during the 1st charge process

State	0.21V [Å] (1st charge)	0.368 V [Å] (1st charge)	0.42 V [Å] (1st charge)	2.5 V [Å] (1st charge)
Na1-O3	2.46850	2.47851	2.46878	2.47720
Na1-O5	2.64033	2.63983	2.64114	2.64322
Na1-O7	2.88481	2.88492	2.88305	2.88805

Average length	2.66454	2.66775	2.66432	2.66949

Table S9 The BVS values based on Na-O bond for the 1st discharge

State	BVS
2.3 V [Å] (1st discharge)	0.28893
1.063 V [Å] (1st discharge)	0.31824
0.11 V [Å] (1st discharge)	0.32379
0.054 V [Å] (1st discharge)	0.32325

Table S10 The fitting results of EIS analysis by Zview software

Cycle	R_s	R_{sei}	R_{ct}	R_{sei}+ R_{ct}	Zw-R	Error [%]
1st	6.087	43.49	9.61	53.10	59.55	1.8461
10th	3.165	10.61	51.43	62.04	1184	8.8136
100th	7.586	26.73	67.17	93.9	5462	1.3616

References

[S1] M. Herklotz, F. Scheiba, M. Hinterstein, K. Nikolowski, M. Knapp, A.-C. Dippel, L. Giebeler, J. Eckert, H. Ehrenberg, *J. Appl. Cryst.* **2013**, *46*, 1117.

[S2] D. Mikhailova, O. M. Karakulina, D. Batuk, J. Hadermann, A. M. Abakumov, M. Herklotz, A. A. Tsirlin, S. Oswald, L. Giebeler, M. Schmidt. J. Eckert, M. Knapp, H. Ehrenberg, *Inorg. Chem.* **2016**, *55*, 7079.

[S3] J. F. Ni, S. D. Fu, C. Wu, Y. Zhao, J. Maier, Y. Yu, L. Li, *Adv. Energy Mater.* **2016,** *6*, 1502568.

[S4] S. Y. Dong, L. F. Shen, H. S. Li,; G. Pang, H. Dou, X. G. Zhang, *Adv. Funct. Mater.* **2016,** *26*, 3703.

[S5] G. B. Xu, L. W. Yang, X. L. Wei, J. W. Ding, J. X. Zhong, P. K. Chu, *Adv. Funct.Mater.* **2016,** *26* , 3349.

[S6] C. J. Wu, Z. G. Wu, X. B. Zhang, R. Rajagopalan, B. H. Zhong, W. Xiang, M. Z. Chen, H. T. Li, T. R. Chen, E. H. Wang, Z. G. Yang, X. D. Guo, *ACS Appl. Mater. Interfaces* **2017,** *9*, 43596.

[S7] Y. S. He, A. Muhetaer, J. M. Li, F. F. Wang, C. Liu, Q. Li, D. S. Xu, *Adv. Energy Mater.* **2017**, 1700950.
